# A reliable method for quantification of splice variants using RT-qPCR

**DOI:** 10.1186/s12867-016-0060-1

**Published:** 2016-03-15

**Authors:** Julia Camacho Londoño, Stephan E. Philipp

**Affiliations:** Experimentelle und Klinische Pharmakologie und Toxikologie, Universität des Saarlandes, 66421 Homburg, Germany

## Abstract

**Background:**

The majority of protein isoforms arise from alternative splicing of the encoding primary RNA transcripts. To understand the significance of single splicing events, reliable techniques are needed to determine their incidence. However, existing methods are labour-intensive, error-prone or of limited use.

**Results:**

Here, we present an improved method to determine the relative incidence of transcripts that arise from alternative splicing at a single site. Splice variants were quantified within a single sample using one-step reverse transcription quantitative PCR. Amplification products obtained with variant specific primer pairs were compared to those obtained with primer pairs common to both variants. The identities of variant specific amplicons were simultaneously verified by melt curve analysis. Independent calculations of the relative incidence of each variant were performed. Since the relative incidences of variants have to add upto 100 %, the method provides an internal control to monitor experimental errors and uniform reverse transcription. The reliability of the method was tested using mixtures of cDNA templates as well as RNA samples from different sources.

**Conclusion:**

The method described here, is easy to set up and does not need unrelated reference genes and time consuming, error-prone standard curves. It provides a reliable and precise technique to distinguish small differences of the relative incidence of two splice variants.

## Background

Ninety-two to ninety-four percent of the human genes express primary RNA transcripts that undergo alternative splicing [1]. This provides an extensive collection of protein isoforms that display different properties and serve a huge variety of functions [2]. This way, alternative splicing is one of the main mechanisms to expand biological diversity. It is a tightly regulated process and splicing errors cause a number of human diseases [3]. RNA splicing is cell type specific and influenced by a number of external factors such us toxins or drugs. A number of splice variants exhibit different drug sensitivities which offers new perspectives for pharmacological intervention [4]. To investigate the significance of splicing and the role of each isoform, a well-founded knowledge of the incidence of alternatively spliced transcripts in a defined cell type or tissue is indispensable.

In the last few years, RNA sequencing has become a valuable tool for the detection of alternative splicing due to increasing capacities, declining costs and newly developed statistical methods [5, 6]. In spite of that, real-time reverse transcription quantitative polymerase chain reaction (real time RT-qPCR) is still regarded as the gold standard to compare the number of mRNA transcripts of different genes [7–10] or alternatively spliced transcripts of a single gene [11, 12]. However, existing methods to quantify the relative ratio of alternative transcripts by RT-qPCR are laborious, error-prone or of limited use.

A recently published method allows the relative quantification of isoforms in two different samples [12]. However, highly different expression levels of each isoform are a prerequisite for its use [12]. This method is applicable to variants that carry unique sequence information each, but not to the majority of variants differing only by the presence or absence of one single exon or a part of it. Finally, it does not allow the quantification of spliced transcripts in a single sample and requires stably expressed reference genes. Therefore, it is only suited for a limited number of applications.

In a pioneer work, Vandenbroucke and collaborators introduced real-time RT-qPCR as an appropriate tool to quantify splice variants [11]. They proposed the use of two primer pairs, each specific for one single variant and they showed that a strategy using a boundary spanning primer is superior to a boundary spanning TaqMan probe for the identification of a spliced isoform. However, the use of boundary spanning primers may still lead to false positives and reduced fidelity because of the high degree of similarity between the exon–exon junction sequences of differentially spliced mRNAs. Furthermore, the strategy did not consider variations of reverse transcription due to different secondary structures of mRNA isoforms. Finally, the method was based on time consuming cDNA standard curves and different amplification efficiencies of the reactions were not considered.

Here we present an improved and reliable method to quantify the ratio of splice variants in a single sample based on RT-qPCR. In addition to two variant specific primer pairs, a further primer pair was introduced. Such control primers anneal to the transcripts of both splice variants, providing an internal control of the procedure. They may flank the splice site, enabling simultaneous identification of both isoforms. Reverse transcription and polymerase chain reaction take place in one single reaction tube (one-step RT-PCR) facilitating the procedure and reducing the risk of technical variations. The new method requires neither an external reference gene nor standard curves. It provides great flexibility regarding the choice of the primers. Therefore, it appears adaptable to most splice events.

## Methods

### Primer design

To test our new method, we designed primers for the cDNA sequences JX644971 and JX644976 of two *Transient Receptor Potential Melastatin 3* (*TRPM3*) variants using the program Primer-BLAST, available online at http://www.ncbi.nlm.nih.gov/tools/primer-blast/. All primers met the requirements of the default settings, which were modified as follows: Their melting temperatures were calculated to 72 ± 0.6 °C. All primers showed a GC content of 40–60 % with a GC clamp of at least two nucleotides. The primer pair specificity was analysed using the reference sequence database of *Mus musculus* (taxid: 10090). All primers showed a maximal self-complementarity of eight nucleotides and a maximal 3′ end complementarity of three nucleotides. Primers had at least four total mismatches to unintended targets, including at least three mismatches within the last four base pairs (bp) at the 3′ end. Primers of each pair derived from separate exons to exclude PCR products of genomic origin. The maximal amplicon size of a primer pair was below 210 bp. Misprimed off-target PCR products had to differ in size by at least 10,000 bp. Furthermore, the annealing regions of all primers were checked for single nucleotide polymorphisms and formation of hairpins using the in silico evaluation tools of the RTPrimerDB database (http://www.rtprimerdb.org [13]). The following HPLC-purified primers were purchased from Eurofins MWG Operon (Germany): control2, forward 5´ TCG CTC GCA GCC AGA TCT TTA TTT A, reverse 5´ GGT ACA ATG TAT TTG AGG GCC CAT GTC; control1, forward 5´ AGC CTG GAA CAG AGT TGA CAT CGC, reverse 5´ TCT GTC CAG GAC TAG GGC ATC CAG; +13, forward 5´ GCA TGC ACC GTT TTC TCA CC ATC, reverse 5´ GGT ACA ATG TAT TTG AGG GCC CAT GTC; Δ13, forward 5´ TGG AAC AGA GTT GAC ATC GCT CG, reverse 5´ TGA GGG CCC ATG TCT TCC ATT TTC.

### Sample acquisition, RNA extraction and quality control

C57BL/6-129/SvJ hybrid mice were killed by cervical dislocation. Their entire brains (n = 3) were immediately isolated, snap frozen in liquid nitrogen, stored at −80 °C and used for experiments within 4 weeks. Choroid plexi isolated from six C57BL/6-129/SvJ hybrids, were shortly rinsed with ice-cold, sterile phosphate buffered saline (PBS). Three of them were pooled (n = 2), snap frozen in liquid nitrogen and stored at −80 °C not longer than 10 weeks. For experiments, frozen samples were immediately resuspended in peqGOLD RNAPure^®^ reagent (Peqlab, Erlangen Germany) and total RNA was extracted following the instructions of the manufacturer. The isolated RNA was further purified by four additional extraction steps using one volume of diethyl ether followed by ethanol precipitation using 0.3 M potassium acetate. For all extraction steps RNase-free/DNase-free plasticware and solutions were used. Isolated RNA samples were dissolved in DEPC treated water, stored in aliquots at −80 °C and thawed only once for one-step RT-qPCR analysis. According to the guidelines for the publication of RT-qPCR experiments [14, 15] the quantity, purity and integrity of all used RNA stocks and dilutions were controlled by spectrophotometry (Nanodrop 1000; Thermo Scientific) and microfluidic analysis using a Bioanalyzer 2100 ([16], Agilent Technologies, Böblingen, Germany). For the microfluidic analysis the Agilent RNA 6000 Nano Kit was used according to the instructions of the manufacturer after decontaminating the electrodes with RNase ZAP (Agilent Technologies, Böblingen, Germany). The different preparations had A_260_/A_280_ values between 1.8–2.1 and A_260_/A_230_ ratios above 2. The preparations showed high RNA integrity with RNA integrity numbers (RIN) between 8.2–8.4 for brain RNA and 9.1–9.2 for choroid plexus RNA.

### Plasmid construction and storage

PCAGGSM2-IRESGFP plasmids [17] containing the cDNA of either variant 1 (GenBank accession JX644976) or variant 2 (GenBank accession JX644971) in the EcoRV site of the polylinker sequence were prepared using the EndoFree Plasmid Maxi Kit (Qiagen, Hilden) and stored in 50 µg aliquots at −20 °C. Plasmid concentrations were measured using the Nanodrop 1000 spectrophotometer. Molar concentrations were calculated using the molecular mass of each plasmid determined with the sequence manipulation suite (http://www.ualberta.ca/~stothard/javascript/dna_mw.html).

### Real-time polymerase chain reaction

RT-qPCR of RNA (30 ng) or quantitative PCR of cDNA samples were performed at least in triplicate at a RotorGene 6000 real-time analyzer (Qiagen, Hilden, Germany). Unless otherwise indicated, 2.5 pM mixtures of the two plasmids described above were used. Unless otherwise indicated, we used the SensiMix™ One-step Kit (Quantace, London, U.K.). In a total volume of 10 µl we added the template, 5 µl SensiMix™ one-step reagent, containing reaction buffer, reverse transcriptase, heat activated DNA polymerase, dNTPs, internal reference, stabilizers and 6 mM MgCl_2_, 0.2 µl SYBR Green solution (50×; Quantace, London, U.K.) and 300 nM of each primer. Then, the following cycling profile was applied: reverse transcription for 30 min at 45 °C (for RNA samples only) followed by incubation for 10 min at 95 °C. For amplification, 30–40 cycles were performed each with incubation at 95 °C for 15 s, followed by 12 s at 68 °C and 15 s at 72 °C. In some cases we used the Express One-Step SYBR GreenER Kit (Life technologies). In a total volume of 10 µl, we added the template, 5 µl SuperMix Universal, 0.25 µl SuperScript Mix and 300 nM of each primer, and then the following cycling profile was applied: 5 min/60 °C (RNA only); 2 min/95 °C; 35–40 cycles of 95 °C/15 s, 62 °C/40 s.

### Calculation of the relative incidence of spliced transcripts

The basic equation of PCR amplification presents the relation of the number of amplicon molecules (N_Cq_) obtained at the quantification cycle (Cq) and the initial number of target molecules (N_0_).

1 $${\text{N}}_{\text{cq}} = {\text{N}}_{0} \cdot 2^{\text{Cq}}$$

Since this equation is valid only for an ideal amplification with a perfect doubling of each amplicon from cycle to cycle, the Cq value was replaced by an efficiency (E, with 1 ≤ E ≤ 2) corrected Cq value (CqE) calculated as recommended [18].

2 $${\text{CqE}} = {{{\text{Cq}} \cdot \log \left( {\text{E}} \right)} \mathord{\left/ {\vphantom {{{\text{Cq}} \cdot \log \left( {\text{E}} \right)} {\log \left( 2 \right)}}} \right. \kern-0pt} {\log \left( 2 \right)}}$$

This led to Eq. (3).

3 $${\text{N}}_{\text{cq}} = {\text{N}}_{0} \cdot 2^{{{\text{cq}} \cdot {{\log \left( {\text{E}} \right)} \mathord{\left/ {\vphantom {{\log \left( {\text{E}} \right)} {\log \left( 2 \right)}}} \right. \kern-0pt} {\log \left( 2 \right)}}}}$$

Unless otherwise indicated, PCR efficiencies of each individual reaction were calculated with the comparative quantitation tool of the RotorGene 6000 analysis software [19]. The comparative quantitation (CQ) method uses the second derivative of raw fluorescence values—i.e. the slope of the amplification curve—to calculate both, the efficiency and the take off point at which the exponential phase of amplification begins [19]. This take off point was used as Cq value [19].

Those data were compared to efficiency values obtained with the LinRegPCR program [20]) or with standard curves using the equation E = 10^−1/M^ with M as slope of the standard curve. Efficiency values of technical replicates obtained by the comparative quantitation or the LinReg tool were averaged to the mean efficiency per amplicon, prior to calculate efficiency corrected C_q_ values. Rearrangement of Eq. (3) provided the mathematical relationship between the initial number of target molecules and the number of amplicons at the quantification cycle.

4 $${\text{N}}_{0} = {{{\text{N}}_{\text{cq}} } \mathord{\left/ {\vphantom {{{\text{N}}_{\text{cq}} } {2^{{{\text{cq}} \cdot {{\log \left( {\text{E}} \right)} \mathord{\left/ {\vphantom {{\log \left( {\text{E}} \right)} {\log \left( 2 \right)}}} \right. \kern-0pt} {\log \left( 2 \right)}}}} }}} \right. \kern-0pt} {2^{{{\text{cq}} \cdot {{\log \left( {\text{E}} \right)} \mathord{\left/ {\vphantom {{\log \left( {\text{E}} \right)} {\log \left( 2 \right)}}} \right. \kern-0pt} {\log \left( 2 \right)}}}} }}$$

**Fig. 1:**
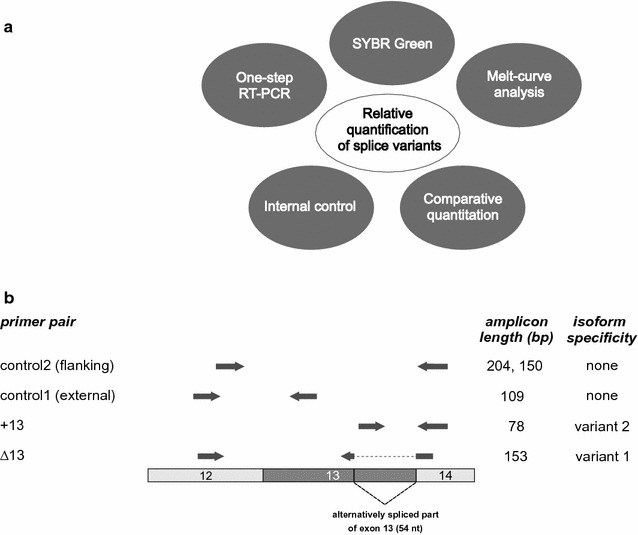
Concept to quantify alternative splice variants. **a** Key elements of the RT-qPCR based strategy for the relative quantification of splice variants. **b** Scheme of the TRPM3 mRNA structure with exon 12–14 and the alternatively spliced part of exon 13. Different primer pairs and their location relative to the TRPM3 mRNA are shown as *arrows*. The length of their amplicons [bp] and their isoform specificity are indicated

For relative quantification, we normalized the number of target molecules of a single splice variant to control amplicons (1 or 2) of transcripts of the very same gene and the very same sample (Fig. 1b). In contrast to unrelated reference genes, which are generally used for normalization, these amplicons provide the most reliable reference possible, since their abundance is strictly linked to the abundance of the splice variant in the given sample. The relative incidence of a single splice variant (RIV) is defined as the initial amount of a single splice variant [N_0_ (var)] relative to the initial amount of the control [N_0_ (con)] and can be presented as

5 $$\begin{aligned} {\text{RIV}} &= {{{\text{N}}_{ 0} \left( {\text{var}} \right)}/ {{\text{N}}_{0} \left( {\text{con}} \right)}} \\&= {{{{{\text{Ncq}}\left( {\text{var}} \right)} /2}^{{{\text{Cqvar}} \cdot \log \left( 2 \right)}} } / {{{{\text{Ncq}}\left( {\text{con}} \right)} / {2^{{{{{\text{Cqcon}} \cdot { \log }\left( {\text{Econ}} \right)} / {\log \left( 2 \right)}}}} }}}}\end{aligned}$$

Since the reactions were performed in parallel at the same PCR instrument the number of both amplicons obtained at the Cq (N_Cq_) was identical. Therefore the equation could be simplified:

6 $$\begin{aligned}{\text{RIV}} &= {{{\text{N}}_{ 0} \left( {\text{var}} \right)} \mathord{\left/ {\vphantom {{{\text{N}}_{ 0} \left( {\text{var}} \right)} {{\text{N}}_{0} \left( {\text{con}} \right)}}} \right. \kern-0pt} {{\text{N}}_{0} \left( {\text{con}} \right)}} \\&= 2^{{{{{\text{Cqcon}} \cdot { \log }\left( {\text{Econ}} \right)} \mathord{\left/ {\vphantom {{{\text{Cqcon}} \cdot { \log }\left( {\text{Econ}} \right)} {\log \left( 2 \right)\text{ - }{\text{Cqvar}} \cdot \log {{\left( {\text{Evar}} \right)} \mathord{\left/ {\vphantom {{\left( {\text{Evar}} \right)} {\log \left( 2 \right)}}} \right. \kern-0pt} {\log \left( 2 \right)}}}}} \right. \kern-0pt} {\log \left( 2 \right)\text{ - }{\text{Cqvar}} \cdot \log {{\left( {\text{Evar}} \right)} \mathord{\left/ {\vphantom {{\left( {\text{Evar}} \right)} {\log \left( 2 \right)}}} \right. \kern-0pt} {\log \left( 2 \right)}}}}}}\end{aligned}$$

Finally, the value was scaled to percentage, to allow comparison of the relative incidence of a single splice variant between different samples:

7 $$\begin{aligned}{\text{RIV}} &= {{{\text{N}}_{ 0} \left( {\text{var}} \right)} \mathord{\left/ {\vphantom {{{\text{N}}_{ 0} \left( {\text{var}} \right)} {{\text{N}}_{0} \left( {\text{con}} \right)}}} \right. \kern-0pt} {{\text{N}}_{0} \left( {\text{con}} \right)}} \cdot 100\left[ \% \right] \\&= 2^{{{{{\text{Cqcon}} \cdot { \log }\left( {\text{Econ}} \right)} \mathord{\left/ {\vphantom {{{\text{Cqcon}} \cdot { \log }\left( {\text{Econ}} \right)} {\log \left( 2 \right)\text{ - }{\text{Cqvar}} \cdot \log {{\left( {\text{Evar}} \right)} \mathord{\left/ {\vphantom {{\left( {\text{Evar}} \right)} {\log \left( 2 \right)}}} \right. \kern-0pt} {\log \left( 2 \right)}}}}} \right. \kern-0pt} {\log \left( 2 \right)\text{ - }{\text{Cqvar}} \cdot \log {{\left( {\text{Evar}} \right)} \mathord{\left/ {\vphantom {{\left( {\text{Evar}} \right)} {\log \left( 2 \right)}}} \right. \kern-0pt} {\log \left( 2 \right)}}}}}} \cdot 100\left[ \% \right] \end{aligned}$$

Since the two different splice variants derive from the same pre-mRNA and are mutually exclusive, their relative incidences to the control amplicon of the same transcript (RIVs) had to add up to 100 %.

8 $${\text{RIV}}_{1} \text{ + }{\text{RIV}}_{2} = 100\,\%$$

This way of analysis provided a unique and easy control for the correctness of the calculation.

### Analysis of the amplification products

The identity of the PCR products and their purity in each sample were controlled after the last amplification cycle by melting curve analysis. In some cases, the results were confirmed by gel electrophoresis. For melting curve analysis, SYBRGreen stained products were kept at 60 °C for 90 s and melted by raising the temperature by 0.3 °C per second up to 95 °C. Products stained with SYBRGreenER were analysed with a similar protocol but with a rate of 0.2 °C per second.

### Statistical analysis

Mean data are given ±SE. Statistical significance of differences between the starting amount of control2 (defined as 100 %) and control1, control2 and variant2 and control2 and variant1 were determined using the unpaired, two-tailed students *t* test (*p < 0.05, **p < 0.01, ***p < 0.001, *ns* not significantly different p > 0.05). All tests were performed using Microsoft Excel.

## Results and discussion

### Strategy of the method

To determine the relative incidence of two splice variants the method of choice should be reliable and as simple as possible. To meet these requirements, our strategy was based on five key elements (Fig. 1a).

1. For the sake of simplicity and robustness, we used one-step RT-PCR with gene specific priming of reverse transcription. In one-step RT-PCR, reverse transcription is carried out in the same tube as the PCR reaction. It is easy to install, involves less error-prone working steps and reduces the risk of contamination [21].

2. We chose SYBR Green staining of DNA amplicons as method of detection. SYBR Green binds to double stranded DNA and is an economical and well-established fluorescent dye for RT-qPCR that can be used for all PCR-products. In contrast to gene specific fluorescent probes, DNA binding dyes do not limit the choice of primer sequences and offer high flexibility for the adaptation of the method to different splice events. Since each amplicon may bind different amounts of dye, producing different fluorescence emission, in principle, an individual proportionality constants “k” for each amplicon is needed for comparison [22]. These amplicon specific constants are unknown and may even change during the course of the reaction as the reporter/DNA ratio changes [22]. In assays using the more common comparative threshold cycle method, this problem is circumvented by comparing the same amplicon from two samples so that these constants are cancelled [23, 24]. This is not applicable to our approach, because two different amplicons are compared within a single sample. However in practice, the influence of unequal binding of SYBR Green to amplicons of different size or GC content appeared to be negligible [25, 26]. Rutlege and Stewart showed for amplicons ranging in size from 100 to 400 bp that the fluorescence intensity generated by SYBR Green was independent of GC content and amplicon size [25]. In line with this finding, Spandidos and coworkers also did not observe any length dependent or AT/GC dependent sequence specificity of SYBR Green when they analyzed purified amplicon DNA [26]. Furthermore and as discussed below, we included an internal control, which readily uncovers experimental errors including errors based on unequal binding of SYBR Green to amplicons.

3. SYBR Green staining also provided the basis for the identification of amplicons by melting curve analysis, which was included as third key element into our strategy. Melting curve analysis is a measurement of the temperature dependent dissociation of two DNA strands during heating. That way, the identity of the PCR product and the absence of off-target amplification products were confirmed.

4. Sample to sample variations of the integrity and purity of the RNA and the use of different primer pairs might strongly affect the efficiency of the amplification process. Therefore, a correction for efficiency of the cycle of quantification (Cq) value is indispensable [23]. We used Cq and efficiency values calculated with the comparative quantification tool (CQ) of the Rotor Gene PCR instrument to determine efficiency corrected Cq values (CqE). The comparative quantification tool is based on the second derivative maximum method and has been proposed as a convenient and accurate way to determine the efficiency and the Cq of PCR reactions [19, 27]. It uses the second derivative of raw fluorescence values—i.e. the slope of the amplification curve—to calculate both, the efficiency and the take off point at which the exponential phase of amplification begins [19].

5. To analyse the frequency of two splice variants by RT-qPCR, in principle, two primer pairs are sufficient [11]. However, local differences of the secondary mRNA structure of the splice variants may cause a disparity of reverse transcription and may distort the result. Furthermore, data might be biased by experimental errors (see above). This illustrates the need of an internal control (Fig. 1a). As a fifth key element of our strategy, we introduced control primer combinations matching a common sequence of both splice variants (Fig. 1a, b). Since the common sequence is independent of splicing, the control primers were used to determine the total frequency of all variants. Their amplicon can be regarded as single but ideal reference (gene) since its rate is invariant and strictly linked to the expression of the isoforms, obviating the need for other, unrelated reference (housekeeping) genes for normalization. Using the amplicons of the control primers for normalization, the relative incidence of each single variant was calculated, individually. Since the sum of alternatively spliced transcripts expressed from one gene is identical to its total number of protein coding transcripts, the relative incidence of the two variants should add up to 100 %. This way, the introduction of a control primer pair and the calculation of relative incidences provided an easy and efficient way to monitor variations of reverse transcription and experimental errors.

As a model to test our approach we chose the *Transient receptor potential melastatin 3* (*Trpm3*) gene. The *Trpm3* gene expresses a number of splice variants, some of which encode ion channel proteins with considerable functional differences [28, 29]. Splicing of 54 nucleotides from *Trpm3* transcripts at the 3´end of exon 13 (Fig. 1b) removes a protein domain that is indispensable for the function of TRPM3 ion channels [29]. However, TRPM3 variants devoid of this region are ubiquitously present in different tissues and cell types [29]. Using a set of two primer pairs for RT-qPCR, we recently showed that their transcripts constitute 2–15 % of the TRPM3 protein isoforms [29].

First, we designed a boundary spanning primer (overlapping the splice site), which hybridized only to the spliced transcript of variant 1 (Fig. 1b). The design of this primer was most delicate due to its limitation to the sequence flanking the splice junction. Special attention was taken to its length and its 3´sequence to avoid mispriming. This boundary spanning primer was combined with a primer located outside the spliced region in an adjacent exon. Using this primer pair named ∆13, one single product of 153 bp specific for variant 1 was expected (Fig. 1b). Second, we designed a primer located within the excised part of the exon to detect non-spliced transcripts of variant 2 (Fig. 1b). Likewise, the primer was combined with a primer located outside the spliced region (primer pair +13) to amplify one single product of 78 bp. Third, we introduced control primers that matched to sequences common to both variants located outside of the spliced region. Both primers of control primer combination 1 were located upstream and close to the spliced region. They amplified identical fragments of 109 bp from both types of transcripts (Fig. 1b). RT-qPCR using these primers was independent of splicing because the sequence and the secondary structure of their target amplicon were identical in both TRPM3 variants. Therefore, this primer pair served as solid internal reference to determine relative incidence values (RIV) of each single variant. To establish the method, these three primer pairs—one specific for each of the variants and one control primer pair—were sufficient. However, we additionally included a second control primer pair that flanked the spliced region (Fig. 1b). This control primer pair 2 provided the opportunity of simultaneous detection of two distinguishable amplicons of 150 bp and 204 bp each specific for the spliced and the non-spliced variant, respectively. If the reverse transcription of one alternative transcript were impeded, the use of this primer pair would lead to miscalculations. Therefore, this control primer pair 2 may serve as internal control only if its efficiency corrected Cq values (CqE) match to those obtained with control primer pair 1.

### Optimization and adjustment of PCR conditions

**Fig. 2:**
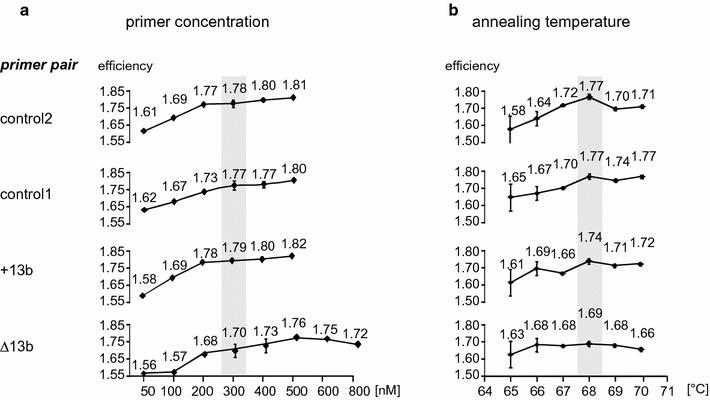
Optimization of primer concentration and annealing temperature. Efficiencies of PCRs were analysed for each primer pair in the presence of different primer concentrations (**a**) or using different annealing temperatures (**b**). A 1:1 mixture of two plasmids each encoding the cDNA of one of the two variants (1.25 pM each) served as template. Mean PCR efficiency (E) values and their SEM of three independent experiments are shown. The best PCR conditions are shaded in *grey*

We tested different PCR cycling parameter such as primer concentration, annealing temperature and dye concentration with a 1:1 mixture of two plasmids encoding the different splice variants. As shown in Fig. 2a, increasing primer concentrations up to 300 nM significantly improved the efficiency of the reactions. At higher concentrations improvements were modest but the risk of primer dimer formation increased substantially (not shown). Therefore, we used 300 nM concentrations of each primer throughout the following experiments. The melting temperature Tm of each primer was calculated to be 72 ± 0.6 °C. We found that the optimal annealing temperature, providing the most efficient amplification of each primer combination, was 68 °C (Fig. 2b).

**Fig. 3:**
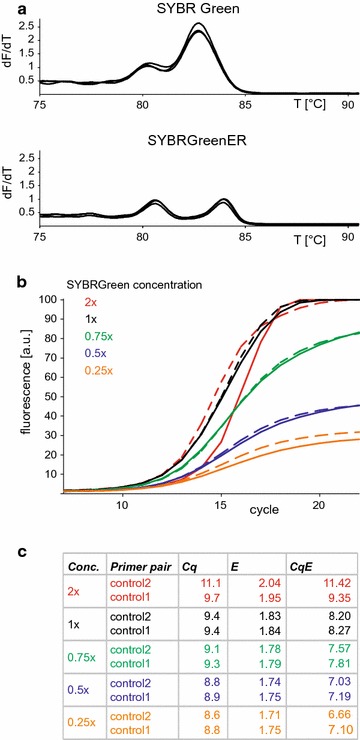
SYBR Green is suitable to analyse alternative splicing by RT-qPCR. **a** Melting curve analysis of the two variant specific fragments amplified with control primers 2 from mRNA transcripts expressed in the choroid plexus of mouse brain in the presence of onefold concentrated (1 ×) SYBR Green or SYBRGreenER. **b** Amplification curves using a 1:1 mixture of two plasmids containing the cDNA of variants 1 and 2 (1.25 pM each) in the presence of different SYBR Green concentrations. Dye concentrations are indicated as multiples of the concentration recommended by the supplier, since it was impossible to obtain detailed information about the molarity of the dye in the reagent. *Dashed lines* show the mean of three reactions using control primer pair 1, *solid lines* using control primer pair 2. **c** The cycle of quantification (Cq) and the PCR efficiency (*E*) of the amplification curves shown in C were obtained from the comparative quantitation tool of the Rotorgene software and efficiency corrected Cq values (CqE) were calculated as described [18]. All curves, the Cq and E values are means of triplicates. Experiments were performed thrice with similar result

Control primers 2, flanking the spliced region, amplified two fragments of different length, each specific for one splice variant (Fig. 1b). Their amplicons could be distinguished by gel electrophoresis or by melting curve analysis. Using these tools, we could easily verify that all primer pairs amplified the desired products without mispriming or primer dimer formation. Using control primer pair 2, we found two discriminable peaks in the melting curve (Fig. 3a). Sequencing of the fragments confirmed their identity (data not shown). SYBRGreenER has been suggested by the manufacturer to be less inhibitory and to provide higher specificity than SYBR Green. Consistently, we detected two clearly separated peaks when we performed RT-qPCR in the presence of SYBRGreenER (Fig. 3a) indicating its superiority for melting curve analysis.

Although SYBRGreenER displayed a better resolution in melting curve analysis, we asked, if the more common dye SYBR Green may be sufficient for our application, or if its inhibitory effect on PCR [30] or its preferential binding to specific DNA sequences [31] may limit its use. Using control primer pair 2 and different SYBR Green concentrations, we determined the cycle of quantification (Cq) and the PCR efficiency (E) and calculated the efficiency corrected Cq (CqE, Fig. 3b, c). Values were compared to those obtained with control primers 1. Increasing dye concentrations led to slightly elevated Cq values, most likely due to inhibition of the PCR by SYBR Green. The steepest amplification curves, the strongest fluorescence signals, and the best efficiencies were obtained with the onefold concentrated dye (1×). The amplification curves obtained with both control primer pairs were highly congruent (Fig. 3b) and revealed very similar Cq, E and CqE values (Fig. 3c), indicating that SYBR Green is sufficient for our application. Furthermore, the experiment already suggested that both primer pairs could serve as control for the quantification of splice events with variant specific primers. However, when we tested the control primers 2 in the presence of the twofold concentrated dye (2×) we observed a strong distortion of the amplification curve leading to efficiency values above 2 and an non-proportional increase of the Cq value (Fig. 3c). Therefore, care should be taken not to exceed the limit of a onefold concentration.

### Range and resolution of detection

**Fig. 4:**
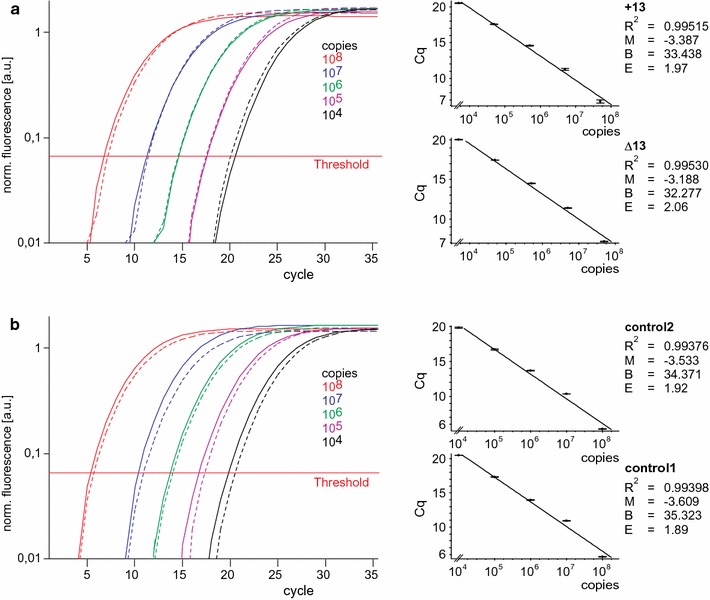
Range of detection of the assay. Amplification curves obtained with primer pair +13 (*solid lines* in **a**) primer pair Δ13 (*dashed lines* in **a**), control primer pair 2 (*solid lines* in **b**) or control primer pair 1 (*dashed lines* in **b**) and different copy numbers of both cDNA templates in equimolar ratio shown as means of triplicates performed thrice with similar results. The corresponding standard curves are presented on the right. Correlation coefficient (*R* ^*2*^), slope (*M*), y-intercept (*B*) and amplification efficiencies (*E*) calculated with the equation E = 10^−1/M^ are indicated

Since the method should be applicable to a broad range of template concentrations, we tested tenfold serial dilutions of two equimolar concentrated plasmids encoding the two different variants. First, we analysed the range of detection with variant specific primer pairs. We obtained highly similar amplification plots and relative standard curves (Fig. 4a), indicating a high specificity of both primer pairs in a range of template concentrations spanning five orders of magnitude. Next, we analysed the two control primer pairs, and again we obtained highly congruent amplification curves (Fig. 4b). The corresponding standard curves displayed linearity with correlation coefficients higher than 0.99. Using the slopes of the standard curves, we calculated amplification efficiencies (E) close to 1.9, confirming the specificity and applicability of both control primer pairs (Fig. 4b).

**Fig. 5:**
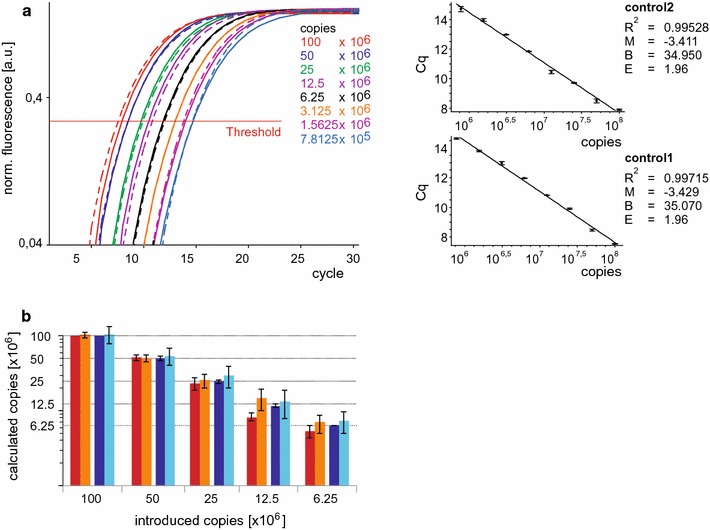
Resolution of the assay. **a** Amplification curves obtained with control primer pair 1 (*dashed lines*) or control primer pair 2 (*solid lines*) and different copy numbers of both templates present in equimolar ratio shown as means of triplicates performed in independent experiments with similar results (n = 5). The corresponding standard curves are shown on the right. Correlation coefficient (*R* ^*2*^), slope (*M*), y-intercept (*B*) and amplification efficiencies (*E*) calculated with the equation E = 10^−1/M^ are indicated. **b** Copies obtained in different reactions (x) with control primer pair 1 or control primer pair 2 at different template concentrations were calculated using the equation: ${\text{copies}} = \left[ {{{{\text{N}}_{0} \left( {\text{X}} \right)} \mathord{\left/ {\vphantom {{{\text{N}}_{0} \left( {\text{X}} \right)} {{\text{N}}_{0} \left( {\text{con2}} \right)}}} \right. \kern-0pt} {{\text{N}}_{0} \left( {\text{con2}} \right)}}} \right] \cdot 10^{8} = 2^{{{{{\text{Cqz}} \cdot { \log }\left( {\text{Ez}} \right)} \mathord{\left/ {\vphantom {{{\text{Cqz}} \cdot { \log }\left( {\text{Ez}} \right)} {\log \left( 2 \right)\text{ - }{{{\text{Cqx}} \cdot { \log }\left( {\text{Ex}} \right)} \mathord{\left/ {\vphantom {{{\text{Cqx}} \cdot { \log }\left( {\text{Ex}} \right)} {\log \left( 2 \right)}}} \right. \kern-0pt} {\log \left( 2 \right)}}}}} \right. \kern-0pt} {\log \left( 2 \right)\text{ - }{{{\text{Cqx}} \cdot { \log }\left( {\text{Ex}} \right)} \mathord{\left/ {\vphantom {{{\text{Cqx}} \cdot { \log }\left( {\text{Ex}} \right)} {\log \left( 2 \right)}}} \right. \kern-0pt} {\log \left( 2 \right)}}}}}} \cdot 10^{8}$ with Cqz and Ez = Cq and E values obtained with control primer pair 2 and 10^8^ introduced copies. The efficiencies introduced into the equation were obtained either from the comparative quantitation tool of the Rotorgene software (*red columns*) or the standard curves shown in A (*blue columns*). *Dark columns* represent reactions using control primer pair 2, *light columns* control primer 1. Mean values ± SE of five independent experiments each performed in triplicate are presented

The method should also enable the detection of small changes of the ratio of variants. Therefore, we analysed the resolution of the method using the control primer pairs 1 and 2 and a twofold serial dilution of a plasmid mixture containing 10^8^ copies of both templates. We obtained overlapping amplification curves for the two primer pairs that were clearly separated for each dilution. The standard curves were almost identical and linear, indicating that even small changes of the template concentration were equally detectable with both control primer pairs (Fig. 5a). The efficiencies determined from these standard curves were used to calculate the numbers of copies that served as templates in the reactions. Regardless of whether we used control primer pair 1 or control primer pair 2, the calculated values correlated with the copy numbers introduced into the reactions (Fig. 5b, blue bars), demonstrating the resolving capacity of the method. The generation of standard curves is labour intensive and relative quantification using standard curve derived efficiencies may provide less reliable result than efficiencies obtained with other methods [19, 20, 32, 33]. Therefore, we repeated the calculation with efficiency values supplied by the comparative quantitation tool of the Rotorgene software (Fig. 5b red bars). Comparing the two procedures, we found no significant differences of the mean values and their corresponding standard errors indicating that both methods of calculation yielded credible data.

**Fig. 6:**
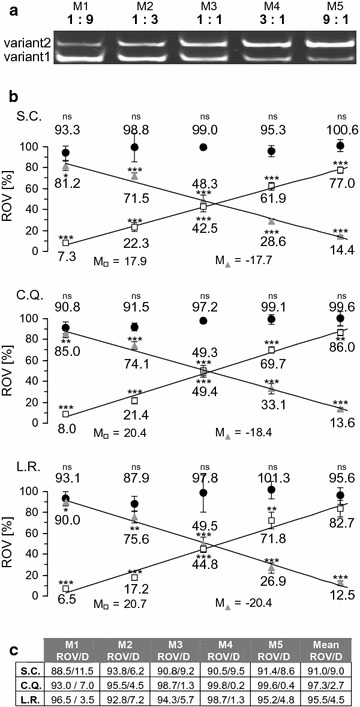
qPCR analysis of different ratios of cDNA variant 1 and 2. **a** Gel electrophoretic separation of amplification products obtained with control primer pair 2 and two plasmids encoding the cDNA of either variant2 or variant1 mixed in five different molar ratios M1– M5 as indicated. **b** Quantification of the different templates in the mixtures M1– M5 presented in A using four different primer pairs. PCR efficiencies (E) were determined using either standard curves (S.C.), the comparative quantitation tool of the Rotor gene software (C.Q.) or using non-baseline corrected data and the LinRegPCR software (L.R). Values for the relative incidences (RIV) of transcripts (x) were calculated using Cq values (Cqx) and E values (Ex) obtained with the control primer pair 1 (filled circles), primer pair +13, (*open squares*) and primer pair Δ13 (*filled triangles*) and the following equation: ${\text{RIV}} = \left[ {{{{\text{N}}_{0} \left( {\text{X}} \right)} \mathord{\left/ {\vphantom {{{\text{N}}_{0} \left( {\text{X}} \right)} {{\text{N}}_{0} \left( {\text{con2}} \right)}}} \right. \kern-0pt} {{\text{N}}_{0} \left( {\text{con2}} \right)}}} \right] \cdot 100\left[ \% \right] = 2^{{{{{\text{Cqcon2}} \cdot {\text{Econ2}}} \mathord{\left/ {\vphantom {{{\text{Cqcon2}} \cdot {\text{Econ2}}} {\log \left( 2 \right)\text{ - }{{{\text{Cqx}} \cdot { \log }\left( {\text{Ex}} \right)} \mathord{\left/ {\vphantom {{{\text{Cqx}} \cdot { \log }\left( {\text{Ex}} \right)} {\log \left( 2 \right)}}} \right. \kern-0pt} {\log \left( 2 \right)}}}}} \right. \kern-0pt} {\log \left( 2 \right)\text{ - }{{{\text{Cqx}} \cdot { \log }\left( {\text{Ex}} \right)} \mathord{\left/ {\vphantom {{{\text{Cqx}} \cdot { \log }\left( {\text{Ex}} \right)} {\log \left( 2 \right)}}} \right. \kern-0pt} {\log \left( 2 \right)}}}}}} \cdot 100\left[ \% \right]$. Data of n = 6 independent experiments for M1–M3, n = 5 independent experiments for M4 and n = 8 independent experiments for M5 are shown with each reaction performed in triplicate. Statistical significance of differences to the starting amount of control2 amplicons (defined as 100 %) were tested using the unpaired two-tailed students *t*-test (*p < 0.05, **p < 0.01, ***p < 0.001, *ns* not significantly different p > 0.05). Linear regression analysis revealed correlation coefficients of at least 0.98 and slopes (*M*) as indicated. **c** Sum of the variant specific RIVs shown in **b** and their difference (*D*) to 100 %

To confirm these findings, we simulated changes of the ratio of the variants and applied the method to mixtures containing the two cDNA templates in ratios of 1:9, 1:3, 1:1, 3:1 and 9:1. Using primer pair 2 we found amplification products of the expected size representing the two different splice variants (Figs. 3a, 6a). We analysed the data using different tools to estimate amplification efficiencies (Fig. 6b). Efficiency corrected Cq values (CqE) obtained with primer pairs +13, Δ13 and control1 were calculated and the starting amounts of their amplicons were normalized to those obtained with control primer pair 2 to determine relative incidence values (RIV) of the transcripts. The RIVs determined with control primer pair 1 were close to 100 %, indicating again that the amplicons of both control primer combinations could be used for normalization. The RIVs obtained with variant specific primers displayed linear relationships with highly similar absolute values of the slopes, indicating that these values indeed mirror the relative incidence of the variants. Furthermore, these RIVs added up to values close to 100 % (Fig. 6c), confirming the principle of the method and its reliability. Using the comparative analysis tool of the Rotorgene software, the sum of variant specific RIVs deviated only slightly from 100 % and the RIV obtained with control primer pair 1 (Fig. 6c). Here, a maximal deviation D_max_ of 7 % was observed in mixture M1 and the mean of the deviations D_mean_ was 2.7 %. Using standard curves (S.C.) or the LinReg Program (L.R.) to calculate amplification efficiencies we found somewhat larger deviations of the sum of variant specific RIVs (S.C.: D_max_ 11.5 %, D_mean_ 9.0 %; L.R.: D_max_ 7.2 %, D_mean_ 4.5 %), indicating that determination of amplification efficiencies using the comparative analysis tool of the Rotorgene software may be not only sufficient but even superior to standard curves.

### Detection and relative quantification of splice variants in tissues

**Fig. 7:**
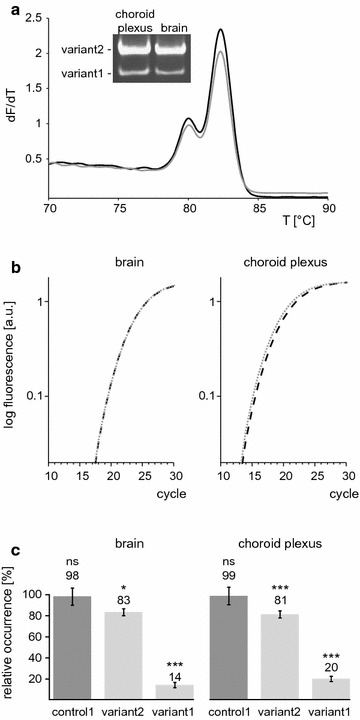
Detection and quantification of alternative transcripts. **a** Melting curve analysis and electrophoretic separation of RT-qPCR products obtained with control primer pair 2 and RNA from mouse brain (*grey line*) and choroid plexus (*black line*). **b** Amplification curves obtained with control primer pair 1 (*dashed lines*) or control primer pair 2 (*solid lines*) and RNA from brain and choroid plexus shown as means of triplicates. **c** Quantification of the relative incidence of TRPM3 splice variants in samples from mouse brain and choroid plexus. PCR efficiencies were determined using the comparative quantitation tool and the relative incidences of variants were calculated as explained in Fig. 6b. Control1 brain represents the mean value ± SEM of eight RIVs obtained in three independent experiments from sample 1, three from sample 2 and two from sample 3 with each reaction performed in triplicate [abbreviated n = 8 (3,3,2)]; variant2 brain [n = 3 (1,1,1)]; variant1 brain [n = 8 (3,2,2)]; control1 plexus [n = 6 (3,3)]; variant2 plexus [n = 11 (6,5)]; variant1 plexus [n = 8 (4,4)]. The statistical significance of the differences to the starting amount of control2 amplicons (defined as 100 %) was tested using the unpaired, two-tailed students *t*-test [*p < 0.05, **p < 0.01, ***p < 0.001, *ns* not significantly different p > 0.05]

We used different RNA preparations from mouse tissues, which were reported to express TRPM3 [28] to test the accuracy and the reliability of the method. Using the control primer pair 2 we initially checked, whether in a relevant tissue both variants were detectable. As shown in Fig. 7a, we found both variants present in total RNA prepared from mouse brain as well as from the choroid plexus of the brain. Next, we compared the amplification curves obtained with the two control primer pairs and found them overlapping (Fig. 7b), indicating that reverse transcription of the variant transcripts was highly similar. Accordingly, we calculated RIVs of 98 and 99 % using control primer pair 1 (Fig. 7c). Using the primer pair +13, we determined a relative incidence of variant 2 of 83 and 81 % in brain and choroid plexus, respectively. Correspondingly, when we performed the analysis with primer pair Δ13, we found that 14 and 20 % of the transcripts encoded variant 1. The Sum of the RIVs of the two variants totalled up to 97 % for brain and 101 % for choroid plexus, confirming the correctness of the data and demonstrating again that the sum of the relative incidences is a useful control of the procedure.

## Conclusions

The presented data confirm our methodological concept and demonstrate that the method is robust and sensitive enough to detect even small changes of the ratio of alternatively spliced transcripts that arise from splicing at a single splice site. However, the method is limited to quantification of such single splice events and does not allow the simultaneous quantification of multiple splice events within one primary transcript. Thus, the method is not recommended to compare isoforms that differ at several regions but rather to analyse the abundance of isoforms with a single defined variation. In contrast to RT-qPCR based analysis of the relative expression of various genes in different samples, our method does not allow to compare the abundance of mRNA isoforms in different samples, but to analyse the ratio of two mutually exclusive transcripts within a single sample, providing important information about splicing frequencies and the relative incidence of the encoded protein isoforms. The method is easy to perform and provides great flexibility regarding the choice of the primer sequences. It can be applied to most splice events and to a broad range of template concentrations and ratios. In addition, the method allows to monitors experimental errors and variations of reverse transcription of the different transcripts and to simultaneously detect two distinguishable, isoform specific products in one single reaction. The method considers different amplification efficiencies of the primer pairs and determines molar ratios of two alternatively spliced transcripts up to 1:9. It is therefore a simple and reliable tool to analyse the incidence and relative changes of two splice variants. Its simplicity and reliability may facilitate its translation to clinical diagnostics of diseases with altered ratio of alternatively spliced RNAs e.g. frontotemporal dementia and Parkinsonism linked to chromosome 17 (FTDP-17) [3].
